# Developing an Internet- and Mobile-Based System to Measure Cigarette Use Among Pacific Islanders: An Ecological Momentary Assessment Study

**DOI:** 10.2196/mhealth.4437

**Published:** 2016-01-07

**Authors:** James Russell Pike, Bin Xie, Nasya Tan, Melanie Dee Sabado-Liwag, Annette Orne, Tupou Toilolo, Steven Cen, Vanessa May, Cevadne Lee, Victor Kaiwi Pang, Michelle A Rainer, Dorothy Etimani S Vaivao, Jonathan Tana Lepule, Sora Park Tanjasiri, Paula Healani Palmer

**Affiliations:** ^1^ School of Community and Global Health Claremont Graduate University Claremont, CA United States; ^2^ Guam Communications Network Long Beach, CA United States; ^3^ Union of Pan Asian Communities San Diego, CA United States; ^4^ University of Southern California Los Angeles, CA United States; ^5^ Tongan Community Service Center Hawthorne, CA United States; ^6^ Pacific Islander Health Partnership Santa Ana, CA United States; ^7^ Samoan National Nurses Association Carson, CA United States; ^8^ California State University, Fullerton Fullerton, CA United States

**Keywords:** Pacific Islander, tobacco use, cigarette use, mobile phone, text message, ecological momentary assessment

## Abstract

**Background:**

Recent prevalence data indicates that Pacific Islanders living in the United States have disproportionately high smoking rates when compared to the general populace. However, little is known about the factors contributing to tobacco use in this at-risk population. Moreover, few studies have attempted to determine these factors utilizing technology-based assessment techniques.

**Objective:**

The objective was to develop a customized Internet-based Ecological Momentary Assessment (EMA) system capable of measuring cigarette use among Pacific Islanders in Southern California. This system integrated the ubiquity of text messaging, the ease of use associated with mobile phone apps, the enhanced functionality offered by Internet-based Cell phone-optimized Assessment Techniques (ICAT), and the high survey completion rates exhibited by EMA studies that used electronic diaries. These features were tested in a feasibility study designed to assess whether Pacific Islanders would respond to this method of measurement and whether the data gathered would lead to novel insights regarding the intrapersonal, social, and ecological factors associated with cigarette use.

**Methods:**

20 young adult smokers in Southern California who self-identified as Pacific Islanders were recruited by 5 community-based organizations to take part in a 7-day EMA study. Participants selected six consecutive two-hour time blocks per day during which they would be willing to receive a text message linking them to an online survey formatted for Web-enabled mobile phones. Both automated reminders and community coaches were used to facilitate survey completion.

**Results:**

720 surveys were completed from 840 survey time blocks, representing a completion rate of 86%. After adjusting for gender, age, and nicotine dependence, feeling happy (P=<.001) or wanting a cigarette while drinking alcohol (P=<.001) were positively associated with cigarette use. Being at home (*P*=.02) or being around people who are not smoking (*P*=.01) were negatively associated with cigarette use.

**Conclusions:**

The results of the feasibility study indicate that customized systems can be used to conduct technology-based assessments of tobacco use among Pacific Islanders. Such systems can foster high levels of survey completion and may lead to novel insights for future research and interventions.

## Introduction

### Assessing Health Disparities among Pacific Islanders

Pacific Islander refers to Chamorros, Marshallese, Native Hawaiians, Samoans, Tongans, and other related groups who share a common origin, culture, and customs. These communities face a wide range of social, economic, and health-related challenges. Educational attainment among Pacific Islanders residing in the United States is low, with only 14.4% obtaining bachelor’s degrees as compared to the national average of 27.9% [1]. Per capita income among Pacific Islanders is US $19,051 whereas the national average is US $27,334 [1]. Smoking-related conditions, such as cardiovascular and respiratory diseases, are disproportionately high among Pacific Islanders [2]. This fact is often overlooked within epidemiological studies that aggregate Pacific Islander data with those of Asian Americans [3]. Disaggregated Asian-Pacific Islander data from the National Adult Tobacco Survey between 2009 and 2010 revealed past month smoking rates of 20.0% compared with 4.7% for Chinese, 5.5% for Asian Indians, 7.2% for Vietnamese, 13.6% for Filipinos, 15.3% for Koreans, and 18.8% for Japanese [4]. Disaggregated data also indicate that Pacific Islanders have smoking rates of 21.5% for males and 18.4% for females compared with respective averages for the general population of 15.7% and 12.8% [5].

One promising assessment technique that may lead to an improved understanding of the factors that contribute to tobacco use among this population is Ecological Momentary Assessment (EMA). Prior tobacco use research has demonstrated that EMA can generate novel insights for future research and interventions [6-26]. Unfortunately, conducting technology-based research among Pacific Islanders involves numerous challenges. Factors that have hindered past research efforts include Pacific Islanders’ broad geographic dispersion and their distrust of academic researchers who often fail to employ culturally-tailored methodologies [27,28]. The objective of the current feasibility study was to overcome these barriers by developing a customized EMA system that facilitated high survey completion rates and fostered new insights into the factors that contribute to cigarette use in this at-risk population.

### Developing an EMA System for Pacific Islanders

EMA is a technique that involves the repeated sampling of participants’ behaviors and experiences in real time within their natural environment [6]. EMA has been used for more than twenty years to measure such behaviors as smoking [6-26,29,30], exercise [31-37], diet [38-42], substance use [43-50], and health information seeking [51]. Historically, these studies have been conducted using electronic diaries [7,9,13-21,24,30,34,39,40,52] that often facilitate survey completion rates greater than 85% [7,13,14,17] but also require extensive in-person training [40,52]. More recently, researchers have reduced respondent burden by utilizing mobile phone apps [53]. However, these programs are often restricted to specific operating systems [11,25,26,31,35,36,50] and are not always programmed to facilitate real-time, remote monitoring of participant responses [31,35,36,50]. Such barriers can be overcome through text messaging [8,44,51,54-56] which is not linked to a specific operating system and typically has higher completion rates. The drawback is that text messaging uses an open-ended format that permits nonstandard responses that can require extensive, time-consuming data cleaning [54]. Another alternative is to employ Internet-based Cell phone-optimized Assessment Techniques (ICAT) to administer online surveys through the Internet browser of Web-enabled mobile phones. This technique is becoming increasingly popular, yet many studies that utilize this approach fail to achieve survey completion rates above 55% [10,29,57]. Moreover, recent ICAT studies of tobacco use [10,12,29] have not capitalized on the unique features offered by this approach, including the ability to use participant responses from earlier in the day to generate tailored survey questions.

**Figure 1:**
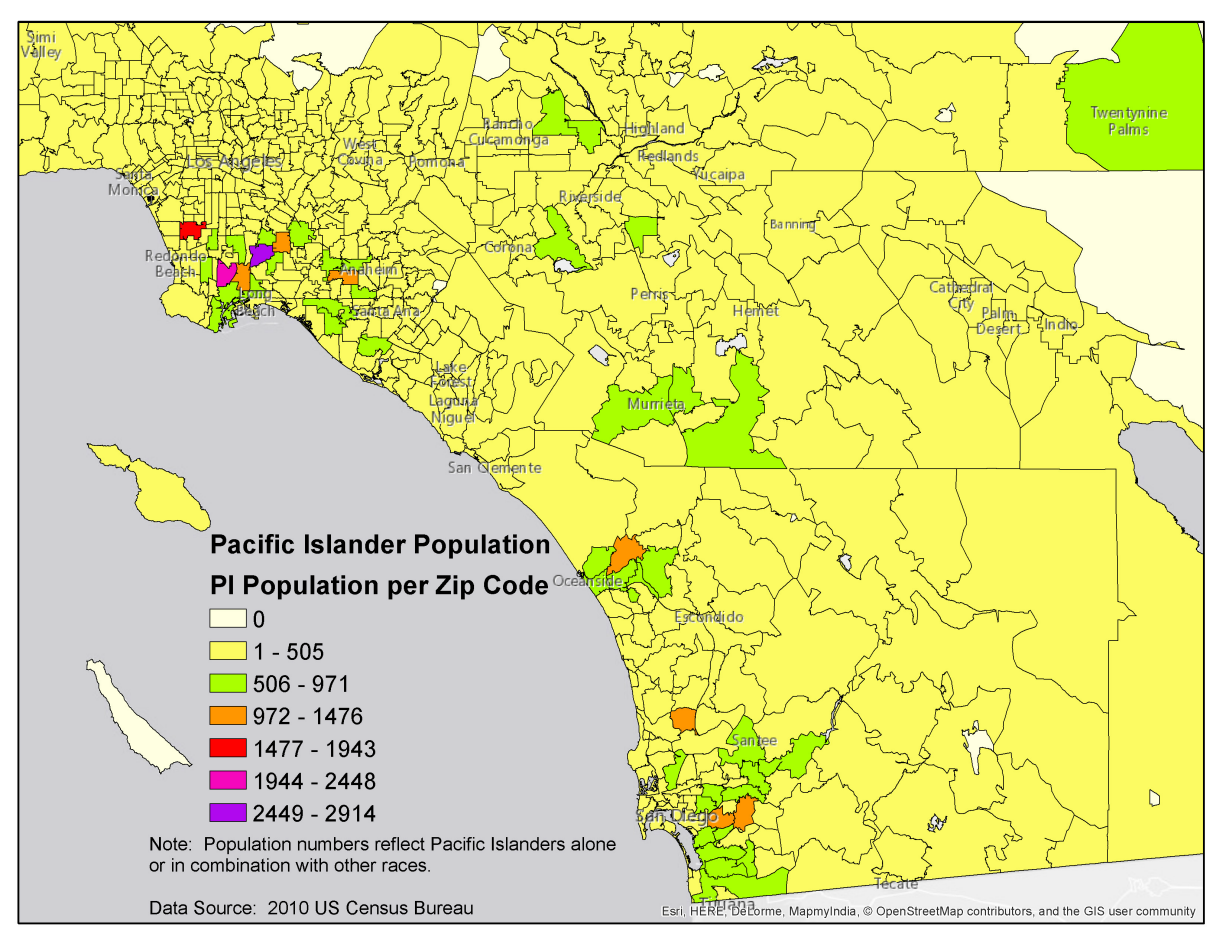
Geographic distribution of Pacific Islanders in Southern California.

In 2011, the Weaving an Islander Network for Cancer Awareness, Research, and Training (WINCART) Center set out to create a customized EMA system that integrated the ubiquity of text messaging, the ease of use associated with mobile phone apps, the enhanced functionality offered by ICAT systems, and the high survey completion rates exhibited by EMA studies that utilized electronic diaries. The WINCART Center is a community-based participatory research [58] consortium that endeavors to reduce cancer health disparities among Pacific Islanders [59] throughout Southern California (see Figure 1). Over a period of several months, members of the consortium collaboratively developed a culturally-tailored system. A feasibility study was then conducted to determine whether young, adult Pacific Islander smokers would be able to effectively utilize the system and whether the resulting data would offer new insights into the intrapersonal, social, and ecological factors associated with cigarette use.

## Methods

### Participants

Using strategies employed in prior studies [60], 5 community-based organizations recruited 208 young adults who self-identified as Chamorro, Marshallese, Native Hawaiian, Samoan, Tongan, or other Pacific Islander. A 12-item screening survey administered in person or over the phone was used to select 61 current smokers who (a) were between 18 and 29 years old, (b) resided in Southern California, and (c) had smoked at least 100 cigarettes in their lifetime. Trained research staff met with each participant at a mutually agreed-upon location. During this meeting, written consent was obtained using a protocol approved by the Institutional Review Boards at Claremont Graduate University and California State University, Fullerton. The participant completed a computer-based questionnaire that assessed their demographic characteristics, tobacco use behavior, and nicotine dependence [61-63]. Research staff also conducted a brief, one-to-one semi-structured interview about the intrapersonal, social, and ecological factors that influenced the participant’s cigarette use. Data from both assessments were used to develop a series of EMA measures for the feasibility study.

Due to the potential burden placed on community coaches tasked with ensuring EMA survey completion during the 7-day feasibility study, the members of the WINCART Center voted to restrict enrollment to 20 participants (see Table 1). These participants were selected to parallel the larger sample in terms of gender, age, tobacco use, and geographic distribution. Of these participants, 60% (12/20) were male. Over half (55%, 11/20) of the participants reported smoking more than 10 cigarettes per day. A mid-point recoding strategy (i.e. 0 for the response “did not smoke,” 0.5 cigarettes for the response “less than 1 cigarette,” 3.5 cigarettes for the response “2-5 cigarettes,” etc) was applied to estimate that an average of 11.8 cigarettes (SD 5.75) were smoked per day per participant.

**Table 1:** Descriptive statistics for demographics and tobacco use from 20 participants.

General characteristics		Male N (%)	Female N (%)
**Ethnic identification**			
	Chamorro	2 (16.7)	0 (0)
	Native Hawaiian	1 (8.3)	0 (0)
	Marshallese	0 (0)	0 (0)
	Samoan	5 (41.7)	2 (25)
	Tongan	4 (33.3)	5 (62.5)
	Other Pacific Islander	0 (0)	1 (12.5)
**Age**			
	18-20	1 (11.2)	0 (0)
	21-23	2 (22.2)	1 (16.7)
	24-26	2 (22.2)	2 (33.3)
	27-30	4 (44.4)	3 (50)
**Education**			
	Less than high school	0 (0)	0 (0)
	High school or GED	8 (72.7)	4 (50)
	Some college/trade school	1 (9.1)	3 (37.5)
	2-year college	1 (9.1)	1 (12.5)
	4-year college or above	1 (9.1)	0 (0)
**Employment status**			
	Employed	7 (58.3)	4 (57.1)
	Unemployed	5 (41.7)	3 (42.9)
**Days smoked in past 30 days**			
	0 days	0 (0)	0 (0)
	1 or 2 days	0 (0)	0 (0)
	3 to 5 days	1 (9.1)	1 (12.5)
	6 to 9 days	0 (0)	0 (0)
	10 to 19 days	0 (0)	0 (0)
	20 to 29 days	3 (27.3)	1 (12.5)
	All 30 days	7 (63.6)	6 (75)
**Cigarettes smoked per day**			
	Less than 1 cig	0 (0)	0 (0)
	1 cig	0 (0)	0 (0)
	2-5 cig	3 (25)	2 (25)
	6-10 cig	2 (16.7)	2 (25)
	11-20 cig	5 (41.7)	4 (50)
	More than 20 cig	2 (16.7)	0 (0)
**FTND nicotine dependence score**			
	Low (<=5)	12 (60)	8 (40)
	High (>=6)	0 (0)	0 (0)
**Lifetime use of alternative tobacco products**			
	Hookah	8 (72.7)	6 (75)
	Cigars	8 (72.7)	4 (50)
	Pipe	4 (36.4)	1 (12.5)
	Smokeless (chew, betel nut, etc.)	4 (36.4)	1 (12.5)
	Cloves	3 (27.3)	0 (0)
	Bidis	1 (9.1)	1 (12.5)
	Kreteks	0 (0)	0 (0)
	Other	0 (0)	1 (12.5)
	None of the above	2 (18.2)	1 (12.5)

### Procedures

**Figure 2:**
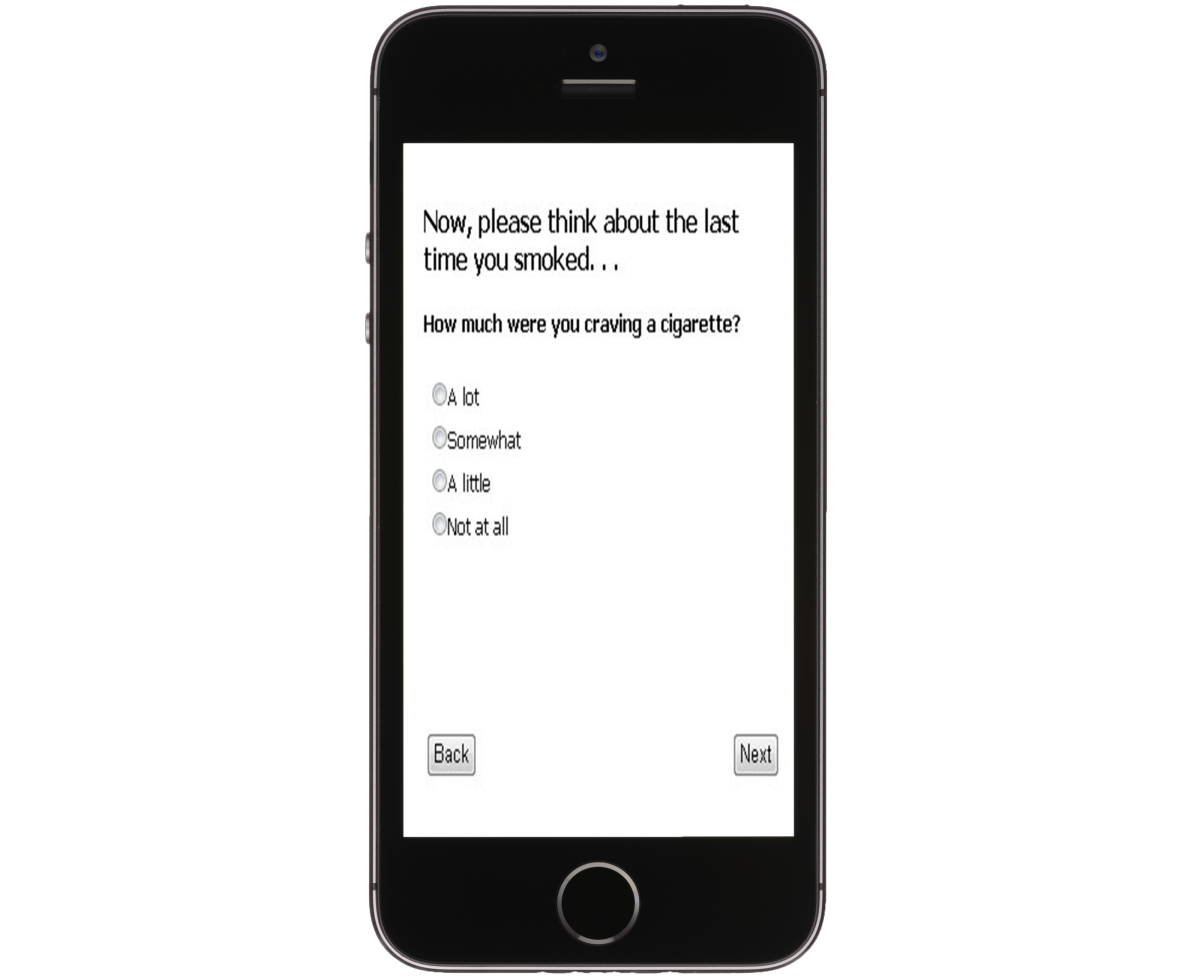
Web-based survey formatted for Web-enabled mobile phones, tablets, and computers.

Each of the twenty participants enrolled in the EMA study attended a one-on-one, follow-up appointment at which the customized system was presented. Surveys were initiated by sending a text message to the participant. This text message contained a link to a secure SQL Server hosting a real-time, Web-based survey system formatted to work on any Web-enabled mobile phone (see Figure 2) as well as any Web-enabled tablet or computer. Participants accessed the system with a self-created username and password. After logging in, the system recognized the participant, recalled the responses entered earlier in the day, and presented a tailored survey.

**Figure 3:**
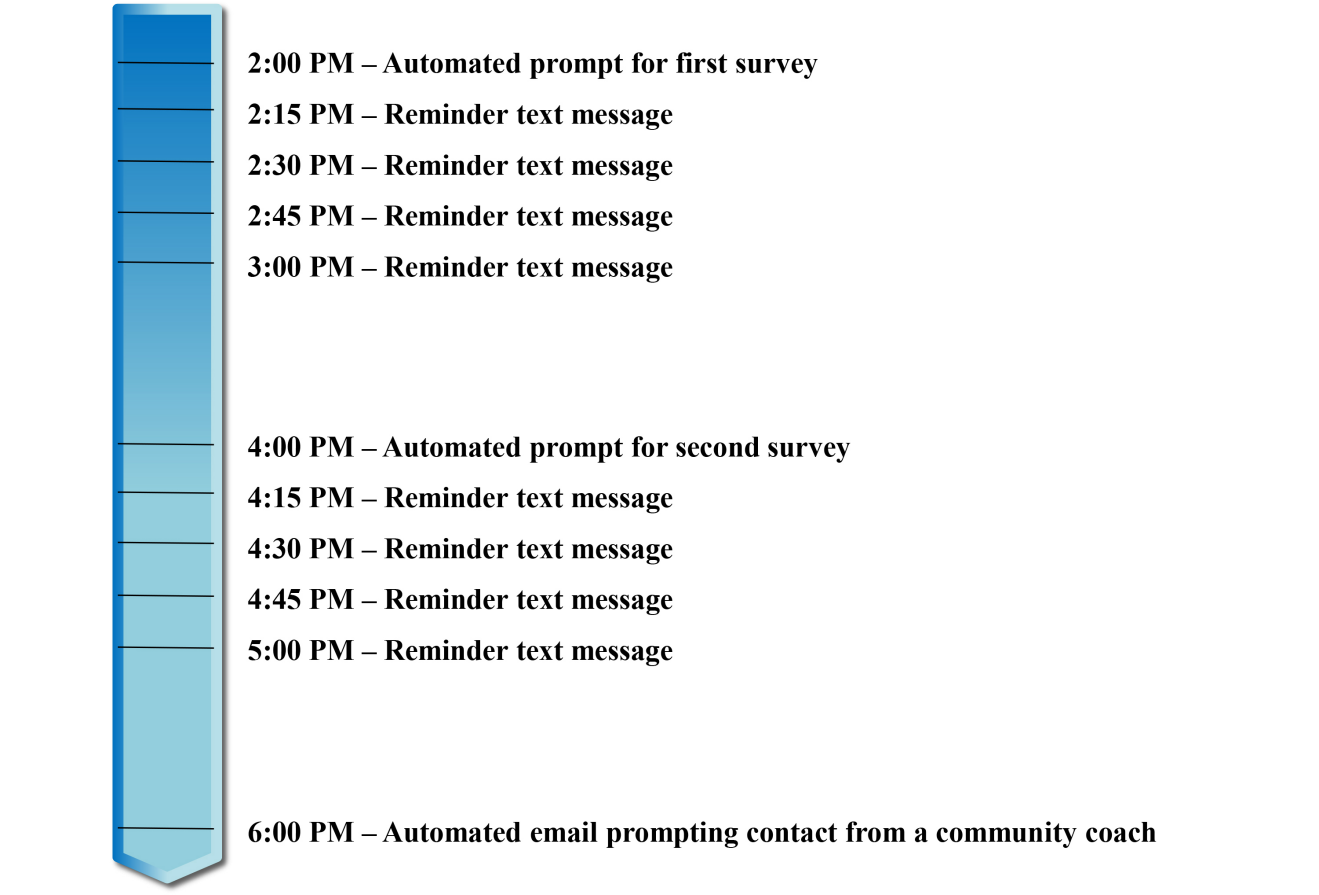
Example timeline of survey reminders.

The Web-based survey was programmed so that if a participant did not complete the first question within 15 minutes of the first text message being sent another text message was delivered. This process was repeated every 15 minutes for up to one hour (see Figure 3). The system then waited an hour before initiating the next survey. If the participant failed to begin a survey after ten text messages, an automated email was sent to a community coach who contacted the participant by phone and reminded the individual to complete the survey. This reminder process was explained to the participant through 8 one-minute, animated videos (see Multimedia Appendices 1-8). These videos used Pacific Islander characters to demonstrate key concepts and were posted online [64] so that the participant could review the materials remotely throughout the study.

After watching the animated videos, the participant selected six consecutive two-hour time blocks during which they would be willing to receive automated text messages each day. These text messages were delivered on even-numbered hours (2:00 PM, 4:00 PM, 6:00 PM, etc.) and asked the participant to report their cigarette use since the last time a survey was completed. Text messages were delivered to a Web-enabled mobile phone either owned by the participant or provided to the participant for the duration of the study. The videos informed the participant that 6 surveys would be administered each day for a period of 9 days. The first 2 days were designed to help the participant acclimate to the process of completing the surveys and to resolve any technical problems encountered. The 7 days after that were the critical test days. At the end of this 9-day period, research staff provided each participant with a US $75 gift card to compensate them for their time and travel.

### Measures

Items for the EMA survey were developed based on data gathered from the computer-based questionnaire and the semi-structured interview. The EMA survey included measures of cigarette use, craving, location, social environment, mood, and rationale for deciding whether or not to smoke. The resulting items were then refined in consultation with the 5 community-based organizations. This process resulted in items that, while unconventional when compared to traditional EMA studies, were more appropriate for the Pacific Islander community.

Participants initially responded to a single question inquiring if they had smoked since the last time they entered a response that day. If they had smoked, the survey asked the number of cigarettes they had (“How many cigarettes have you had since {time of last entered response}?”). This question was designed so that all responses in a single day could be tabulated and compared to self-report measures of daily cigarette use. The question also facilitated the analytic approach which focused on the average number of cigarettes smoked across time blocks within each day. A follow-up question determined when the participant smoked (“When did you smoke?”) utilizing four response categories (“<30 mins ago,” “30-60 mins ago,” “60-90 mins ago,” and “90-120 mins ago”). This question did not ascertain the number of cigarettes smoked in each 30-minute sub-block but was instead used to gauge how much time had passed since the last instance in which the participant smoked. The remaining questions asked the participant to reflect on this instance (“Now, please think about the last time you smoked...”).

After recalling the last instance in which they smoked, the participant answered one question about the extent to which they craved a cigarette beforehand (“How much were you craving a cigarette?”). Responses were rated on a 4-point Likert scale ranging from “1=Not at all” to “4=A lot” (see Figure 2). The participant then reported their location (“Where were you?”) by selecting among the response options “Home,” “Work,” “School,” “At a restaurant/bar,” “In a car,” “Around church,” and “Other (Please type in).” The next question asked the participant to classify their social environment (“Who were you with when you last smoked?”) using the response options “Alone,” “People who are smoking,” “People who are NOT smoking,” and “Other (Please type in).” The survey then used a “Check all that apply” format to assess the participant’s mood (“How were you feeling?”) using the categories “Happy,” “Sad,” “Angry,” “Stressed out,” “Anxious,” “Bored,” “Relaxed,” “Fine,” and “Other (Please type in).” The final question asked the participant to reflect on why they had chosen to smoke (“Why did you smoke?”) and respond using a “Check all that apply” format comprised of the categories “Someone offered me a cig,” “Someone was smoking around me,” “Wanted a cigarette while drinking alcohol,” “Wanted a cigarette while drinking coffee,” “Wanted a cigarette after eating,” “To eat less,” “To relax or calm down,” “Have a good time/celebrate,” “To concentrate/focus,” and “Other (Please type in).”

### Analysis

Cigarette use was reported in 534 time blocks. The analysis focused on the total and average number of cigarettes smoked during each time block. Repeated assessments of factors associated with the most recent cigarette consumed were aggregated by taking the average of these assessments across time blocks within each day. This produced a two-level hierarchical analysis dataset with data on each day (i.e. level-1 data) nested within individual participants (i.e. level-2 data). Multilevel models with both fixed and random effects were then used to quantify between and within subject variability across repeated measurement points [16,65,66].

Variables were created for the associational analysis. These variables included the total number of cigarettes smoked during time blocks per day, the proportion of time blocks per day in which 4 or more cigarettes were smoked, and the proportion of time blocks per day in which the participant reported an intrapersonal, social, or ecological factor related to cigarette use. Multilevel regression models were conducted using SAS Proc Mixed procedure with the participants’ age, gender, and nicotine dependence being adjusted in the analysis as covariates [67]. Analysis results with raw data are presented and consistent parameter estimates were obtained with multiple imputation analysis from SAS PROC MI and MIANALYZE [68].

## Results

### Survey Completion and Reported Cigarette Use

Surveys in which the participant failed to respond to all questions within two hours of the first text message prompt were classified as missed. In total, 20 participants completed 720 surveys from 840 prompted survey time blocks, representing a prompt-based survey completion rate of 86%. Nineteen (95%) participants completed all surveys on the first five days. Sixteen (80%) participants completed all surveys on the sixth day and fifteen (75%) participants completed all surveys on the seventh day. Participants reported smoking in 535 (74%) of the 720 two-hour time blocks with completed surveys. The average total number of cigarettes smoked per day was 13.96 (SD 9.19) which is 18.3% higher than the recoded estimates reported in the computer-based questionnaire.

### Factors Associated With Cigarette Use

For each time block in which smoking was reported, participants had an average of 3.07 cigarettes (SD 1.44). Home was the most common smoking location and attempting to relax was the most commonly cited reason for choosing to smoke (see Table 2).

**Table 2:** Proportions of responses per day during time blocks in which smoking was reported.

Question	Response option	Mean of proportion (SD)
**Where were you?**		
	At home	0.61 (0.37)
	In a car	0.20 (0.28)
	At work	0.13 (0.28)
	Around church	0.07 (0.23)
	At restaurant/bar	0.05 (0.12)
	At school	0.01 (0.07)
**Who were you with when you last smoked?**		
	Alone	0.54 (0.35)
	With people who are not smoking	0.41 (0.36)
	With people who are smoking	0.23 (0.29)
**How were you feeling?**		
	Fine	0.51 (0.39)
	Relaxed	0.37 (0.37)
	Happy	0.18 (0.30)
	Stressed out	0.16 (0.31)
	Bored	0.10 (0.17)
	Anxious	0.09 (0.20)
	Angry	0.03 (0.13)
	Sad	0.02 (0.11)
**Why did you smoke?**		
	To relax or calm down	0.55 (0.35)
	Wanted a cigarette after eating	0.26 (0.29)
	Someone was smoking around me	0.15 (0.26)
	Someone offered me a cig	0.11 (0.25)
	To concentrate/focus	0.11 (0.24)
	Have a good time/celebrate	0.09 (0.20)
	Wanted a cigarette while drinking alcohol	0.05 (0.14)
	Wanted a cigarette while drinking coffee	0.03 (0.09)
	To eat less	0.03 (0.10)

After adjusting for gender, age, and nicotine dependence, analyses indicated that a one score increase of craving resulted in 3.8 more cigarettes smoked per day on average (*P*<.001). In addition, feeling happy (*P*<.001) or wanting a cigarette while drinking alcohol (*P*<.001) (see Table 3) were positively associated with the total number of cigarettes smoked during time blocks. Being at home (*P=*.02) or being around people who are not smoking (*P=* .01) were negatively associated with the total number of cigarettes smoked during time blocks. These associations persisted when participants reported smoking 4 or more cigarettes (see Table 4). Several other associations were also identified when the proportion of time blocks per day in which 4 or more cigarettes were smoked was used as the outcome variable in the analysis. Positive associations included feeling angry (*P=*.05) and wanting a cigarette while drinking coffee (*P=*.01). Negative associations included feeling bored (*P=*.02) and wanting to eat less (*P=*.02).

**Table 3:** Associations with total number of cigarettes smoked per day.

Question	Response option	β	SE	*P*
**Where were you?**				
	At home	-0.72	0.31	.02
	In a car	-0.02	0.42	.96
	At work	0.49	0.44	.27
	Around church	0.79	0.85	.36
	At restaurant/bar	0.17	0.71	.82
	At school	1.51	1.16	.20
**Who were you with when you last smoked?**				
	Alone	0.54	0.29	.06
	With people who are not smoking	-0.76	0.28	.01
	With people who are smoking	0.65	0.35	.07
**How were you feeling?**				
	Fine	0.31	0.29	.30
	Relaxed	0.19	0.37	.60
	Happy	1.41	0.37	< .001
	Stressed out	0.13	0.43	.76
	Bored	-0.85	0.54	.12
	Anxious	0.9	0.55	.11
	Angry	0.88	0.71	.22
	Sad	0.03	0.84	.97
**Why did you smoke?**				
	To relax or calm down	0.41	0.32	.22
	Wanted a cigarette after eating	0.26	0.39	.50
	Someone was smoking around me	0.15	0.43	.72
	Someone offered me a cig	0.72	0.47	.13
	To concentrate/focus	-0.52	0.53	.33
	Have a good time/celebrate	0.46	0.55	.41
	Wanted a cigarette while drinking alcohol	2.31	0.63	<.001
	Wanted a cigarette while drinking coffee	1.85	1.08	.09
	To eat less	-1.32	1.25	.29

**Table 4:** Associations with proportion of time blocks per day with four or more cigarettes.

Question	Response option	β	SE	*P*
**Where were you?**				
	At home	-0.19	0.08	.02
	In a car	0.06	0.11	.59
	At work	0.09	0.11	.40
	Around church	0.07	0.20	.74
	At restaurant/bar	0.00	0.19	.99
	At school	0.91	0.29	.003
**Who were you with when you last smoked?**				
	Alone	0.09	0.07	.23
	With people who are not smoking	-0.17	0.07	.03
	With people who are smoking	0.17	0.09	.06
**How were you feeling?**				
	Fine	0.01	0.08	.92
	Relaxed	0.08	0.09	.42
	Happy	0.29	0.10	.003
	Stressed out	0.14	0.11	.19
	Bored	-0.32	0.14	.02
	Anxious	0.24	0.14	.10
	Angry	0.37	0.18	.05
	Sad	-0.01	0.22	.95
**Why did you smoke?**				
	To relax or calm down	0.09	0.08	.28
	Wanted a cigarette after eating	0.12	0.10	.23
	Someone was smoking around me	-0.04	0.11	.75
	Someone offered me a cig	0.09	0.12	.44
	To concentrate/focus	-0.21	0.14	.12
	Have a good time/celebrate	0.19	0.14	.19
	Wanted a cigarette while drinking alcohol	0.38	0.17	.03
	Wanted a cigarette while drinking coffee	0.77	0.27	.01
	To eat less	-0.73	0.31	.02

## Discussion

### Implications for Future Research and Interventions

This is the first study, of which we are aware, in which a customized EMA system assessed factors associated with tobacco use among young, adult Pacific Islanders. Prior EMA studies of tobacco use that utilized electronic diaries reported prompt-based survey completion rates of 65% [15], 68% [30], 75% [9], 88% [17], 89% [14], 90% [7], and 91% [13]. ICAT studies that relied upon the use of Web-based surveys administered through mobile phones reported prompt-based survey completion rates of 50% [29], 52% [57], 55% [10], 69% [42], and 83% [12]. The survey completion rate of the current study was 86% (720/840), suggesting that young, adult Pacific Islanders were able to effectively use the customized EMA system and that this form of assessment holds promise for future research. The survey completion rate also indicates that remotely accessible standardized training videos (see Multimedia Appendices 1-8) may serve as a reasonable alternative to extensive in-person training and technical support—especially for populations spread out over a large geographic region.

Prior research has suggested that while EMA measures sometimes mirror the results of recall measures they often gather data with less noise and greater sensitivity [6]. The assessment of daily cigarette use provides additional evidence to support this conclusion. Self-report measures administered at the outset of the study indicated that each participant smoked an average of 11.8 (SD 5.75) cigarettes per day. In contrast, the EMA system cataloged an average of 13.96 (SD 9.19) cigarettes smoked per day. The health implications of this are notable in that they translate to an estimated 769 additional cigarettes, or approximately 38.5 packs, per year per participant.

An analysis of the EMA data also offers new insight into the intrapersonal, social, and ecological factors associated with cigarette use among young, adult Pacific Islanders. Previous studies have identified the home as a place where smoking frequently occurs [13]. Yet, the fact that Pacific Islanders in the current study smoke fewer cigarettes than average at home suggests that this location could serve as a constructive environment for practicing smoking cessation techniques. Past EMA research has also highlighted how smokers are more likely to have a cigarette when in the presence of other smokers [11-15]. However, the protective influence of nonsmokers on Pacific Islander tobacco use suggests that such individuals may function as a deterrent that can be relied upon in social situations where smoking is prevalent. Future smoking cessation interventions tailored for Pacific Islanders may choose to test these concepts by providing educational materials that promote the use of nonsmoking social networks when attempting to quit using tobacco products.

A technological innovation within the current study, the creation of tailored survey questions based on participant responses entered earlier in the day, hints at new opportunities for additional research. Future studies may choose to capitalize on this feature by administering brief, tailored surveys to multiple, linked individuals within the Pacific Islander community each time one individual reports smoking behavior in a prior assessment. Such investigations may lead to an improved understanding of friend and familial perceptions of Pacific Islander tobacco use, which previous research has suggested is highly relevant in explaining both past and current smoking among Native Hawaiian youth [69].

The correlation between increased tobacco craving and increased cigarette use parallels previous findings that cravings precipitate smoking [9] and that higher levels of craving at one time point within a day tend to produce more smoking at the next time point [8]. Similarly, other studies have identified significant positive associations between alcohol consumption and tobacco use [52] and coffee consumption and tobacco use [13]. These similarities suggest that components of existing evidence-based interventions that address these factors may be highly effective for young, adult Pacific Islanders. The results for mood are more ambiguous. Some studies suggest negative affect is predictive of smoking [8,15] while others find no prospective relationship with either positive or negative affect [13,14]. Unfortunately, the measure utilized in the current study does not delineate whether the mood indicated preceded cigarette use or was a direct outcome of it. The correlation between tobacco use and happiness may therefore be the participant experiencing higher positive affect before or after smoking [16]. The inconclusive nature of this finding exposes the need for additional research within this population.

### Limitations

There are several limitations to the current study. Perhaps the most relevant is the small sample size and homogeneity of the participants. Specifically, the generalizability of the sample to other Pacific Islander communities may be limited by the fact that all participants were identified via nonprobability sampling. Moreover, key subgroups, including female Chamorros, Native Hawaiians, and Marshallese, were not represented nor were Pacific Islanders over the age of thirty. The small sample size also prohibited analytic techniques that have generated valuable insights in prior EMA research. Examples include explorations of how situational and mood factors differ between light and heavy smokers [12] and analyses that reveal situational covariates that influence smoking patterns [17].

Another limitation within the current study is the focus on regular cigarettes. With the advent of electronic cigarettes, as well as the common usage of alternative forms of tobacco within the Pacific Islander community [4], the range of behavior measured by future tobacco use studies may need to be broadened. Within the current sample, lifetime hookah use was reported at 73% for males (8/11) and 75% (6/8) for females (see Table 1). This is significantly greater than the averages for the general population of 42% for current male smokers and 23% for current female smokers [5] and suggests that future research should explore the different factors that may be associated with the use of alternative tobacco products.

An additional limitation is that all surveys were initiated on even-numbered hours and only assessed factors associated with the most recent cigarette smoked. Future studies should consider initiating surveys at random intervals within time blocks to avoid potential time-based covariates. Such methods will help clarify the causal relationship between cigarette use and the factors associated with it. Nevertheless, future interventions may still consider applying the current findings even if their causal relationship remains unclear [70,71].

### Conclusions

This feasibility study provides new insights on factors that contribute to cigarette use among young, adult Pacific Islanders. It suggests new directions for conducting advanced, technology-based research aimed at understanding tobacco use within the Pacific Islander community and offers new possibilities for crafting culturally-tailored interventions, which past research has demonstrated is often more effective within this population [72]. Given the disproportionately high smoking rates among Pacific Islanders [5], these types of research and intervention efforts are sorely needed to address the health disparities evident within this population.

## Appendix

Multimedia Appendix 1 Introduction to the ecological momentary assessment study.

Multimedia Appendix 2 Using an iPhone.

Multimedia Appendix 3 Accessing a Web-based survey with an iPhone.

Multimedia Appendix 4 Completing a Web-based survey with an iPhone.

Multimedia Appendix 5 Accessing a Web-based survey after receiving a text message.

Multimedia Appendix 6 Selecting times to receive text message survey reminders.

Multimedia Appendix 7 Receiving text message survey reminders.

Multimedia Appendix 8 Contacting technical support.

## Abbreviations

EMA: Ecological Momentary Assessment
ICAT: Internet-Based Cell Phone-Optimized Assessment Technique
NCI CRCHD: National Cancer Institute Center to Reduce Cancer Health Disparities
SQL: Structured Query Language
WINCART: Weaving an Islander Network for Cancer Awareness, Research, and Training
